# Transactions of the Medical Association of Southern Central New York, at the Eighth Annual Meeting, Held at Homer, June, 1854

**Published:** 1854-09

**Authors:** 


*Transactions of the Medical Assosiation of Southern Central New York, at the eighth Annual Meeting, held at Homer, June,* 1854.

This association comes out with a very neat volume of its transactions for the year, as fair and clean in its typography as it is valuable in its contents.

We know of no voluntary associated effort that deserves so much credit, or is so worthy of imitation as this. Made up of the profession in the counties of Chemung, Schuyler, Tioga, Broome, Tompkins, and Cortland, its members are within visiting distance, and possess common interests. Names such as Hyde, Green, and Hawley, familiar to medical ears, are found among its active men; while its written communications are furnished by very many contributors, who give evidence of very high scientific attainment. Comparisons are invidious; but we are frank to say that the State Medical Society published a volume last spring far inferior to this in scientific merit.

The address of the president, (Prof. Hyde,) had the advantage of being delivered to a large audience of non-professional hearers, who, we hope, profited by its manly vindication of the claims and position of legitimate mediicine, as well as its exposition of the immoral tendencies of quackery.

Of the remainder of the transactions, we have only time to mention the essay on "Opium and the Sulphate of Quinine as Remedial Agents," by Dr. Nelson Nivison, as a very intelligent and logical *resumé* of the more recent theories of remedial action. H.

---

"Dr. Charles Mayor, late of Lausanne, Switzerland, presented the subject of the localization of baths according to a method which he describes in a memoir which he published in Paris, in 1844, entitled *Localization des Baines et de L'Application de Froid et de la Chaleur sur les diverses parties du corps humain.*

"On motion, the thanks of the Association were tendered to Dr. Mayor for his instructive and interesting exhibition of apparatus."—*From Trans. Med. Assoc. of Central Southern New York.*

For the substance of Dr. Mayor's memoir the reader is referred to Prof. Hamilton's translation of Amussat, on the use of Water in Surgery, published in this Journal. On pages 152, '3 and '4 of vol. 7, may be found a full *resumé* of Dr. Mayor's method.

Dr. Mayor, as may be inferred from the preceding extract, is now in this country, having fled hither on account of political disturbances in which his patriotism had involved him. He is now engaged in agriculture, at Berkshire, Tioga Co., in this state. Dr. M. has a distinguished family connection. His father, M. Mathias Mayor, was the well known surgeon of the hospital of Lausanne; while M. Louis Agassiz is his cousin.

The revolutions of Europe, if they fail in their objects, have at least the effect of sending to this country many men of high scientific merit. Here a cordial welcome should be given them as some compensation for the evils of exile and separation from father-land. There should be no Know-Nothings in the Republic of Science.

We hope to be able to present, at some future time, a translation of Dr. Mayor's essay. H.

